# Prevalence and Risk Factors Associated with Suicide Ideation and Attempts in Korean College Students

**DOI:** 10.4306/pi.2008.5.2.86

**Published:** 2008-06-30

**Authors:** Hong-Seok Lee, Sukil Kim, Inyoung Choi, Kyuong-Uk Lee

**Affiliations:** 1 College of Medicine, The Catholic University of Korea, Seoul, Korea.; 2 Department of Preventive Medicine, College of Medicine, The Catholic University of Korea, Seoul, Korea.; 3 Department of Psychiatry, Uijeongbu St. Mary's Hospital, College of Medicine, The Catholic University of Korea, Seoul, Korea.

**Keywords:** Suicide ideation, Suicide attempts, Risk factors, College students

## Abstract

**Objective:**

Suicide is a leading cause of death in college age students. Identification of the associated risk factors has important implications for how to prevent and respond to this population; however, few studies have been performed on this topic in this age group. The purpose of this study was to evaluate the prevalence and risk factors associated with suicide ideation and attempts in college students.

**Methods:**

Three hundred sixty-eight college students participated in this cross-sectional observational study. The recent (over two weeks) suicide ideation and lifetime suicide attempts were defined according to Moscicki's suicide behavior index. Sociodemographic variables were assessed and psychopathology measured using the Beck Depression Inventory, the Bipolar Spectrum Diagnostic Scale and the Alcohol Use Disorders Identification Test. A hierarchical multiple logistic regression analysis was used to identify the significant risk factors related to suicide ideation and attempts.

**Results:**

The two-week prevalence of suicidal ideation was 9.8%, and the lifetime prevalence of suicide attempts was 3.3%. The univariate analysis showed that students who had more severe depression (p<0.001), a higher probability for bipolar disorder (p<0.001) and decrement of academic achievement (p<0.005) were more likely to have suicide ideation. Those with factors such as severe depression (p<0.05), a higher probability of bipolar disorder (p<0.001), a low socioeconomic status (p<0.001), who lived alone (p<0.01), and were female (p<0.05) had a higher risk for suicide attempts. The most important predictors of suicide ideation, by the logistic regression analysis, were depression, probability for bipolar disorder and academic achievement, and the risks identified for suicide attempts were socioeconomic status and probability of bipolar disorder.

**Conclusion:**

Suicide ideation and attempts were common in college students. The results of this study suggest that early identification and management of mood disorders and other sociodemographic risk factors may have implications for intervention and prevention.

## Introduction

The environmental and social factors unique to college students may be characterized as a "transition" period in life. This transition occurs during a brief period of time on many levels including social, academic, psychological, and existential. This major life transition as well as specific risk factors may exacerbate existing psychological difficulties or trigger new ones that can ultimately lead to suicide.

According to a report on the causes of death, suicide was a leading cause of death among people aged 20-29 years in Korea.^1^ The suicide mortality rate was reported as 12.4 persons out of 100,000 for college students aged 20-24; whereas, the rate was 15.3 persons out of 100,000 for people aged 25-29 in 2005.^1^ The prevalence of suicide ideation ranged from approximately 9-12% and was up to 62% in some studies;^2^-^4^ the risk for suicide attempts has been reported to be from 1.8-5%.^3^,^5^ A recent study showed that the lifetime prevalence of suicide ideation was substantial, as high as 39.2%, and that 3.0% of Korean college students had attempted suicide.^6^ Despite the high rate of suicide in this population, the risk factors associated with suicide ideation and attempts have not been studied in college students as a distinct group from the general population.^7^-^11^ Risk factors for suicide ideation and attempts have been reported to include many domains: psychopathology, social and educational disadvantage, childhood and family adversity, individual and personal vulnerabilities, exposure to stressful life events and circumstances, and social, cultural and contextual factors. Research has shown consistent risk factors such as mental disorders and a history of psychopathology.^9^,^12^,^13^ The mood disorders (depression and bipolar disorder) are by far the most common psychiatric conditions associated with suicide. Depression is an important risk factor for suicide in college students, as it is for people in general.^2^,^6^,^14^,^15^ In addition, there is a strong association of bipolar disorder with suicidal ideation and attempts.^16^,^17^ Bipolar disorder preceding depression could be a risk factor for suicide in adolescence as well as college students. However, only one study has been performed evaluating this population.^18^ Another psychiatric disorder associated with suicide is alcoholism. Several studies have reported that in the general population as well as in college students, alcohol dependence is a risk factor for suicide ideation and attempts.^19^-^23^

When studying the risk factors for suicide in college students, academic achievement may be an important factor to consider. Most college students are under pressure to get good grades to ensure future success; thus, academic pressure is likely a significant source of psychological stress for many college students.^24^,^25^ Due to slow economic growth, the overflow of college graduates and the need for experienced workers, the current Korean college students may experience greater stress with regard to job prospects than previous generations. While the association between school/academic stressors and suicide ideation has been well documented among adolescents,^26^-^28^ it has not been investigated in college students. Only a few studies, performed in other countries, have shown a relationship between suicide and academic problems in college students.^29^-^31^ However, no studies have been performed examining this relationship among Korean college students.

Sociodemographic variables such as gender and socioeconomic status may also be important variables to consider when evaluating suicide risks. The medical literature shows that men and women behave differently with respect to suicide. For example, completed suicide rates are higher for men than for women and, by contrast, the rates of suicide attempts are higher for women than for men.^32^ However, for college students, some studies have reported no gender difference for suicide ideation.^33^,^34^

A socially disadvantaged background characterized by features such as low socioeconomic status (SES), low income and poverty has been suggested to be a risk factor for suicide.^35^ A recent systematic review suggested an inverse association, with lower rates of suicide in higher SES groups in the general population;^35^ a Korean study had results consistent with this review.^36^ Financial difficulties, in college students, can increase the students' level of anxiety and depression, which may lead to increased suicide behavior.^37^ However, no prior study has examined the association between suicide and socioeconomic status in Korean college students.

While the presence of risk factors is associated with an increased risk of suicide, protective factors can moderate or lower this risk. Social resources, such as family support, can be protective and a negative predictor of suicide attempts.^38^ Many studies have reported that young people from families with histories of parental separation or divorce have an increased risk for suicide.^39^,^40^

Given the unique psychosocial environment and high rates of suicide ideation and attempts among college students, a better understanding of the risk factors associated with suicide and suicide ideation is needed to provide the appropriate interventions to prevent suicide in this group of young adults. Therefore, the objectives of this study were 1) to study the prevalence of suicide ideation and attempts, 2) to examine the risk factors associated with suicide ideation and attempts, and 3) to identify the predictors of suicide ideation and attempts in Korean college students.

## Methods

### Subjects

College students from five universities in Seoul, Gyeonggi province and Pohang province, participated in this cross-sectional study. A total of 368 students completed self-administered questionnaires. All subjects participated in the survey voluntarily. The psychopathology of the participants was measured by self-rating scales including the Beck Depression Inventory (BDI), the Bipolar Spectrum Diagnostic Scale (BSDS) and the Alcohol Use Disorders Identification Test (AUDIT). The sociodemographic risk factors examined were gender, years in college, socioeconomic status, living with parents, and changes in academic achievement.

### Measurements

#### Suicide Ideation and Attempts

Suicide ideation and attempts were defined by Moscicki's suicide behaviors model.^41^ Suicidal ideation was defined as any suicidal thought within the past two weeks and suicide attempts were defined as any attempt over a lifetime. The two-week time frame for suicide ideation was chosen to reduce recall bias. However, since a suicide attempt itself was considered a traumatic behavior, recall bias was assumed to be minimal and the lifetime prevalence was therefore assessed.

#### Beck Depression Inventory

The BDI consists of 21 items that are based on attitudes and symptoms that Beck observed to be common among depressed patients and uncommon among patients who are not depressed.^42^ The items of the BDI include emotional, behavioral and somatic symptoms. The ratings ranged from 0 to 3 points on the basis of the severity of symptoms. The range of the total scores was from 0 to 37. The scores of 10-15, 16-23 and over 24 indicated mild, moderate and severe depression, respectively.^43^ A Korean translated version was used for this study.^44^

#### Bipolar Spectrum Diagnostic Scale

The BSDS was originally designed to detect milder symptoms of bipolar spectrum in outpatients. The first part of the scale is a paragraph containing 19 positive valence sentences describing many of the symptoms of bipolar disorder. The second part of the BSDS is a simple multiple-choice questionnaire, asking subjects to rate how well a story describes them. There are four possible answers from which to choose; "This story fits me very well, or almost perfectly" (corresponding to 6 points), "this story fits me fairly well" (corresponding to 4 points), "this story fits me to some degree" (corresponding to 2 points), and "this story does not describe me at all" (corresponding to 0 points). The total scores were interpreted as '0 to 6; highly unlikely'; '7 to 11; low probability'; '12 to 19; moderate probability'; or '20 to 25; high probability'.^45^

#### Alcohol Use Disorders Identification Test

The AUDIT has been increasingly used as a screening tool in a variety of evaluations for hazardous or harmful drinking habits. The 10-item questionnaire covers three fields including alcohol consumption, drinking behavior and alcohol-related problems. The response to each question was scored from 0 to 4, with a maximum of 40 points. A score of eight or more indicated hazardous and harmful alcohol use, as well as possible alcohol dependence.^46^,^47^ The Korean translated version was used for this study.^48^

### Analysis

The statistical analysis was performed using Windows SAS 9.0 (SAS Institute Inc., Cary, NC, USA). The univariate analysis was performed using the χ^2^ test (or with continuity correction) to explore the relationship between the sociodemographic variables and psychopathology with suicide ideation and attempts. A two-tailed significance of p<0.05 was defined to be statistically significant. A hierarchical multiple logistic regression analysis followed to identify the risk factors associated with suicidal ideation and attempts. For this regression model, variables that showed significant differences in the univariate analysis were entered manually by one of the coinvestigators.

## Results

Among the respondents, the two-week prevalence of suicidal ideation was 9.8% (36/368) and the lifetime prevalence of suicide attempts was 3.3% (12/368). Most subjects had a middle socioeconomic status (95.4%), were freshmen (81.5%) and there were more males than females (61.1%:38.9%). Table 1 shows the general characteristics of the subjects and the results of the univariate analysis.

The univariate analysis showed that those subjects with a more severe depression were more likely to have suicide ideation [χ^2^ (3, n=368)=50.93, p<0.001]. As the probability of the bipolar disorder increased, the risk for suicide ideation also increased [χ^2^ (2, n=365)=27.45, p<0.001]. Students who experienced a decrement in their academic achievement were more likely to have suicide ideation than those who did not [χ^2^ (1, n=368)=8.78, p<0.005].

The risk factors associated with suicide attempts were then explored. The risk factors related to psychopathology such as a more severe depression or a higher probability of the bipolar disorder were significantly associated with suicide attempts [χ^2^ (3, n=368)=8.14, p<0.05, χ^2^ (2, n=365)=21.67, p<0.001]. In addition, the analysis showed that a low SES was significantly associated with a higher risk for suicide attempts [χ^2^ (2, n=368)=32.39, p<0.001]. Females were more likely to attempt suicide than males [χ^2^ (1, n=368)=4.04, p<0.05]. Furthermore, students living alone were at greater risk for suicide attempts than those living with their parents [χ^2^ (1, n=368)=8.10, p<0.01].

A hierarchical multiple logistic regression analysis was employed to predict the probability that students would have suicide ideation and attempts. The risk factors that showed significance, by the chi-square test, for suicide ideation or attempts were used for the logistic regression model. The predictors selected were depression, probability of bipolar disorder, SES, living with parents, academic achievement, and gender.

Table 2 shows the logistic regression coefficient, Wald statistic, odds ratio, and its confidence interval for each of the risk factors for suicide ideation after adjusting for other factors. When depression was added first, the model significantly improved [χ^2^ (1, n=365)=36.47, p<0.001]. By adding the probability for bipolar disorder, in addition to depression, the model improved further [χ^2^ (1, n=365)=13.04, p<0.001]. Then, after adding academic achievement, in addition to the other two factors, the model showed additional improved [χ^2^ (1, n=365)=4.50, p<0.05]. However, after adding the risk factors including living with parents, SES and gender, showed no additional improvement to the model. The odds ratio for depression indicated that students with depressive symptoms (a BDI score over 10) were 8.88 times more likely to have suicide ideation than those without depressive symptoms, after adjusting for other factors. Students who had a higher probability for bipolar disorder (a BSDS score over 7) were about 4.59 times more at risk for suicide ideation than those who had a highly unlikely probability for bipolar disorder. Among the sociodemographic variables, only decrement of academic achievement predicted an increased risk for suicide ideation in the model (odds ratio 2.26).

Table 3 shows that a low SES and a higher probability for bipolar disorder were significant predictors of suicide attempts. Adding the SES significantly improved the model, [χ^2^ (1, n=368)=13.21, p<0.001]. When the probability of the bipolar disorder was added to the model, in addition to SES, the model improved further [χ^2^ (1, n=365)=33.07, p<0.001]. Students who were in a low SES, compared to middle and high SES students had an increased risk for suicide attempts (odds ratio 69.27). A one point increase in the probability scores for the bipolar disorder increased the odds ratio for suicide attempts 1.33 fold.

## Discussion

The results of this study showed that among Korean college students the prevalence of suicide ideation, during the past two weeks, was 9.8% and that of lifetime suicide attempts was 3.3%. A recent study on Korean college students reported that the lifetime prevalence of suicide ideation was 39.2% and that of suicide attempts was 3%, which is similar to the findings of this study.^6^ The prevalence reported varies among different studies; 1.8% for suicide attempts in one Chinese study,^5^ 9.5% for suicide ideation and 1.5% for attempted suicide within the last school year in the USA.^2^ Another study reported that 10% of college students had serious suicide ideation.^23^ Even though we cannot directly compare the prevalence of this study with that of the other studies because of the different characteristics of the samples and the duration of suicide behavior evaluated, the findings of this study are consistent with prior studies that showed a high prevalence of suicide behavior among college students.

The results of the present study showed that psychopathology including the severity of depression and a higher probability for bipolar disorder were associated with both suicide ideation and attempts. We found that depression was a strong predictor for suicide ideation. Students who had depression (a BDI score over 10) were 8.88 more likely to have suicide ideation, after adjusting for other factors. This finding is consistent with previous studies on college students.^2^,^6^,^14^,^15^,^18^ College students, especially freshmen, most of the subjects in this study were freshmen, are a group particularly prone to stress due to the transitional nature of college life.^49^,^50^

In addition, a higher probability for bipolar disorder was a risk factor for suicide ideation and attempts, consistent with the findings of a prior study.^18^ Students who had low, medium and high probability for bipolar disorder (a BSDS score over 7) were 4.59 times more likely to have suicide ideation than those with a highly unlikely probability (a BSDS score less than 7). The analysis also showed that a one point increase in the BSDS score increased the odds ratio for suicide attempts by 1.33 times. Bipolar disorder is a chronic, intermittent illness that is associated with high morbidity and mortality.^51^ In addition, patients with bipolar disorder often have comorbid psychiatric conditions such as substance abuse, anxiety disorders, attention-deficit/hyperactivity disorder, and eating disorders.^52^ The coexistence of other Axis I disorders with bipolar disorder can mask the clinical features in this age group and complicates the psychiatric diagnosis and treatment; this can result in an increased burden of the illness on the patients, family members and treating clinicians. Although bipolar disorder consists of recurring episodes of mania and depression, patients spend more time depressed than manic. Bipolar depression is often undiagnosed or misdiagnosed as unipolar depression, resulting in incorrect or inadequate treatment.^53^ Thus, strategies for early screening and inclusion among the differential diagnosis of mood disorders are important factors to consider for the college student age group.

Contrary to our expectations, the harmful use of alcohol was not a risk factor for suicide behavior in college students. This finding is in contrast to the findings reported previously that showed an association between suicide and alcohol-related problems such as alcohol dependence, alcohol abuse and acute alcohol ingestion.^19^-^23^ One possible reason for this negative finding may be the characteristics of the study population. Research has shown that suicide is a late outcome of alcoholism and, therefore, the typical alcoholic suicide was associated with a depressed, elderly, married man whose suicide was precipitated by some interpersonal loss, job loss, legal difficulties, or financial trouble.^54^ However, the subjects in the present study were mostly young college students having less than a moderate level of alcohol problems based on the AUDIT scores.^47^ Suicide among alcoholics less than 40 years of age is uncommon.^54^ College suicides are characterized by depressed, quiet, socially isolated young people who do not abuse alcohol or drugs.^55^

In the present study, a decrement in academic achievement was associated with suicide ideation; sociodemographic factors such as low SES, female gender and living alone were associated with suicide attempts. The association between academic stress and suicide ideation among college students has been reported previously in other countries.^29^-^31^ However, although this academic stress-suicide ideation link may be even stronger in Korea, given the familial and cultural demands for academic excellence, there have been no studies conducted in Korea on college students. The results of the present study suggest that a decrement in academic achievement might increase suicide ideation among college students. A low academic performance was significantly associated with high BDI scores. In addition, the hierarchical multiple regression analysis suggested that a decrement of academic achievement was a significant predictor of suicide ideation. The results of a previous study suggested that depression partially mediated the relationship between academic stress and suicide ideation.^27^ Thus, the implementation of programs to help college students experiencing academic difficulties in addition to identification and intervention of students with depression might help reduce suicide.

The findings of this study suggested that a low SES was a risk factor and a strong predictor of suicide attempts. This result is consistent with previous studies that showed that a low SES was associated with high rates of suicide and morbidity and mortality resulting from a high prevalence of mental disorders or health risk behaviors.^11^,^56^ The results of the present study also showed that the low SES group scored significantly higher on the BDI scores compared to the middle and high SES groups. A prior study reported that financial difficulties contributed significantly to depression among college students.^37^ Therefore, students experiencing financial difficulties should be offered access to mental health programs.

Gender differences in suicide rates are well known among the general population.^32^ In the present study, the college women reported suicide ideation and attempts more often than did the men; however, only the suicide attempts showed a statistically significant difference with regard to gender (Table 1). This finding is consistent with a previous study that reported higher suicide behavior in women than in men during college^6^ and in the general population.^57^ However, the present findings differ from prior reports in that previous studies found an almost equal number of males and females expressed some form of suicide ideation or attempts.^33^,^34^,^58^ Gender differences in adaptive cognitive processes and coping skills may account for these discrepancies.^34^

Sociological and family-related factors may also be implicated in college student suicide. The family can be an important source of comfort during stressful periods. Thus, living alone separated from family may leave the student without an immediate support system. In the present study, living alone increased the risk for suicide attempts in the college students. This result suggests that facilitating access to support resources might help reduce the risk for suicidal behavior among college students. In addition to the family, friends are also an important source of support; one report showed that support from friends may be an even greater resource than from the family.^59^ Therefore, evaluating the support system of students, including friends, may further provide information that can help prevent suicide.

Previous studies have reported that suicide ideation is a risk factor for suicide attempts in the general population.^60^ However, suicide ideation was not a predictor for suicide attempts among the college students. In the present study, we assessed suicide ideation based on the presence or absence of suicide ideation over the past two weeks. However, some studies have shown that the severity or variability of suicide ideation might predict suicide attempts. For example, Beck et al. suggested that suicide ideation, at its most severe manifestation, was a risk factor for eventual suicide.^61^ Another study suggested that the variability of suicidal ideation provided better predictive power, for suicide attempts, than did the intensity or duration.^62^

Our results were limited in that we could not ascertain the severity, duration and variability of suicide thoughts or the seriousness of the intent underlying a suicide attempt. Because of the small sample size, the results should be interpreted with caution, as the presence of a type 2 error cannot be excluded. In addition, the psychopathology was evaluated by self-reported measures, thus, the results can not be considered psychiatric diagnoses. Finally, other risk factors such as childhood and family adversity, impulsivity and exposure to recent stressors were not evaluated and need further investigation.

In conclusion, mood disorders and other sociodemographic factors might be associated with an increased risk for suicide ideation and attempts in some Korean college students. The results of this study suggest that reducing suicidal ideation and attempts requires a multi-faceted approach including mental health care services, academic support systems and counseling centers for employment to improve the prospects of employment for students. If appropriate programs are available to support the mental health and coping mechanisms of college students at risk, then the rate of suicide may improve.

## Figures and Tables

**TABLE 1:**
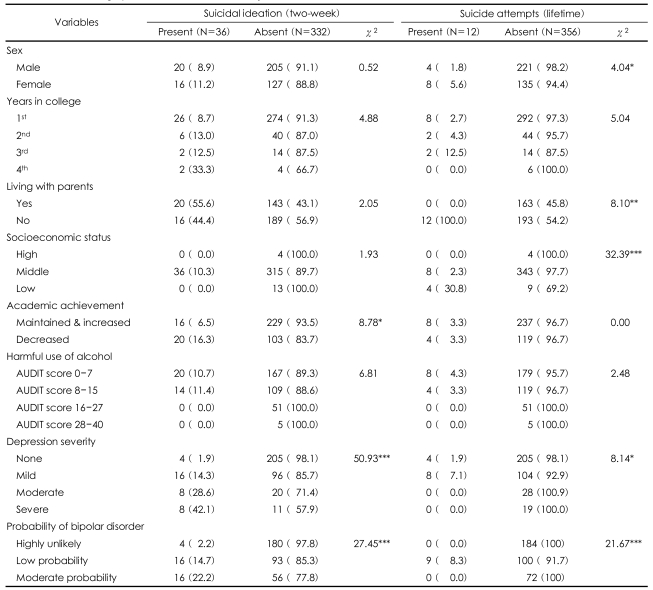
Sociodemographic characteristics of the subjects, N (%)

^*^p<0.05, ^**^p<0.01, ^***^p<0.001. AUDIT: Alcohol Use Disorder Identification Test

**TABLE 2:**
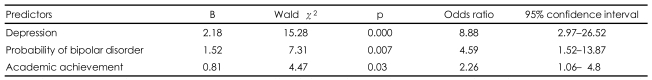
Multiple logistic regression analysis predicting suicidal ideation from clinical and sociodemographic variables

Depression: Beck Depression Inventory Score >10. Probability of bipolar disorder: Bipolar Spectrum Diagnostic Scale Score >7. Academic achievement: decreased vs. increased or maintained

**TABLE 3:**
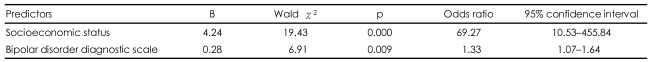
Multiple logistic regression analysis predicting suicide attempts from clinical and sociodemographic variables

Socioeconomic status: low vs. middle and high. Bipolar disorder diagnostic scale: continuous score

## References

[1] (2008). 2005 Annual report on the morbidity rate of suicide.

[2] Kisch J, Leino EV, Silverman MM (2005). Aspects of suicidal behavior, depression, and treatment in college students: results from the spring 2000 national college health assessment survey. Suicide Life Threat Behav.

[3] Westefeld JS, Homaifar B, Spotts J, Furr S, Range L, Werth JL (2005). Perce-ptions concerning college student suicide: data from four universities. Suicide Life Threat Behav.

[4] Schweitzer R, Klayich M, McLean J (1995). Suicidal ideation and behaviours among university students in Australia. Aust N Z J Psychiatry.

[5] Xu HL, Xiao SY, Feng SS, Chen XX (2004). Risk factors for suicide attempt among college students at Central South University. Zhonghua Liu Xing Bing Xue Za Zhi.

[6] Roh MS, Jeon HJ, Lee HW, Lee HJ, Han SK, Hahm BJ (2007). Suicide-related behaviors among the college students. J Korean Neuropsychiatr Assoc.

[7] Yen YC, Yang MJ, Yang MS, Lung FW, Shih CH, Hahn CY (2005). Suicidal ideation and associated factors among community-dwelling elders in Taiwan. Psychiatry Clin Neurosci.

[8] Toros F, Bilgin NG, Sasmaz T, Bugdayci R, Camdeviren H (2004). Suicide attempts and risk factors among children and adolescents. Yonsei Med J.

[9] Beautrais AL (2000). Risk factors for suicide and attempted suicide among young people. Aust N Z J Psychiatry.

[10] Johansson SE, Sundquist J (1997). Unemployment is an important risk factor for suicide in contemporary Sweden: an 11-year follow-up study of a cross-sectional sample of 37,789 people. Public Health.

[11] Oksuz E, Malhan S (2005). Socioeconomic factors and health risk behaviors among university students in Turkey: questionnaire study. Croat Med J.

[12] Park HS, Schepp KG, Jang EH, Koo HY (2006). Predictors of suicidal ideation among high school students by gender in South Korea. J Sch Health.

[13] Spann M, Molock SD, Barksdale C, Matlin S, Puri R (2006). Suicide and African American teenagers: risk factors and coping mechanisms. Suicide Life Threat Behav.

[14] Lee H, Kim M (2007). A pathmodel for self-identity and hopelessness to suicidal ideation of college students. Kor J Youth Stud.

[15] Sohn JN (2007). Discriminating Power of Suicidal ideation by Life Stress, Coping Strategy, and Depression in College Students. J Korean Acad Psych Mental Health Nurs.

[16] Rihmer Z, Kiss K (2002). Bipolar disorders and suicidal behaviour. Bipolar Disord.

[17] Jamison KR (2000). Suicide and bipolar disorder. J Clin Psychiatry.

[18] Rihmer ZZ (2007). Suicide risk in mood disorders. Curr Opin in Psychiatry.

[19] Murphy GE, Wetzel RD (1990). The lifetime risk of suicide in alcoholism. Arch Gen Psychiatry.

[20] Sher L (2006). Alcoholism and suicidal behavior: a clinical overview. Acta Psychiatr Scand.

[21] Lejoyeux M, Huet F, Claudon M, Fichelle A, Casalino E, Lequen V (2008). Characteristics of suicide attempts preceded by alcohol consumption. Arch Suicide Res.

[22] Sher L, Sperling D, Stanley BH, Carballo JJ, Shoval G, Zalsman G (2007). Triggers for suicidal behavior in depressed older adolescents and young adults: do alcohol use disorders make a difference?. Int J Adolesc Med Health.

[23] Brener ND, Hassan SS, Barrios LC (1999). Suicidal ideation among college students in the United States. J Consult Clin Psychol.

[24] Hashim IH (2003). Cultural and Gender Differences in Perceptions of Stressors and Coping Skills: A Study of Western and African College Students in China. Sch Psychol Int.

[25] Bush HS, Thompson M, Van Tubergen N (1985). Personal assessment of stress factors for college students. J Sch Health.

[26] Juon HS, Nam JJ, Ensminger ME (1994). Epidemiology of suicidal behavior among Korean adolescents. J Child Psychol Psychiatry.

[27] Ang RP, Huan VS (2006). Relationship between academic stress and suicidal ideation: testing for depression as a mediator using multiple regression. Child Psychiatry Hum Dev.

[28] Chung HK, Ahn OH, Kim KH (2003). Predicting Factors on Youth suicide Impulse. Kor J Youth Stud.

[29] Hawton K, Haigh R, Simkin S, Fagg J (1995). Attempted suicide in Oxford University students, 1976-1990. Psychol Med.

[30] Hawton K, Simkin S, Fagg J, Hawkins M (1995). Suicide in Oxford University students, 1976-1990. Br J Psychiatry.

[31] Kirmayer LJ, Malus M, Boothroyd LJ (1996). Suicide attempts among Inuit youth: a community survey of prevalence and risk factors. Acta Psychiatr Scand.

[32] Kochanek KD, Murphy SL, Anderson RN, Scott C (2004). Deaths: final data for 2002. National Vital Statistics Reports.

[33] Langhinrichsen-Rohling J, Arata C, Bowers D, O'Brien N, Morgan A (2004). Suicidal behavior, negative affect, gender, and self-reported delinquency in college students. Suicide Life Threat Behav.

[34] Ellis JB, Lamis DA (2007). Adaptive characteristics and suicidal behavior: a gender comparison of young adults. Death Stud.

[35] Rehkopf DH, Buka SL (2006). The association between suicide and the socioeconomic characteristics of geographical areas: a systematic review. Psychol Med.

[36] Hong SC, Kim MD, Lee S (2003). Suicide risk in relation to social class: a national register-based study of all suicide in Korea, 1999-2001. Health and Social Science.

[37] Andrews B, Wilding JM (2004). The relation of depression and anxiety to life-stress and achievement in students. Br J Psychol.

[38] Perkins DF, Hartless G (2002). An Ecological Risk-Factor Examination of Suicide Ideation and Behavior of Adolescents. J Adolescent Res.

[39] Gould MS, Shaffer D, Fisher P, Garfinkel R (1998). Separation/divorce and child and adolescent completed suicide. J Am Acad Child Adolesc Psychiatry.

[40] Brent DA, Perper JA, Moritz G, Liotus L, Schweers J, Balach L (1994). Familial risk factors for adolescent suicide: a case-control study. Acta Psychiatr Scand.

[41] Mościcki EK, O'Carroll P, Rae DS, Locke BZ, Roy A, Regier DA (1988). Suicide attempts in the Epidemiologic Catchment Area Study. Yale J Biol Med.

[42] Beck AT, Ward CH, Mendelson M, Mock J, Erbaugh J (1961). An inventory for measuring depression. Arch Gen Psychiatry.

[43] Steer RA, Brown GK, Beck AT, Sanderson WC (2001). Mean Beck Depression Inventory-II scores by severity of major depressive episode. Psychol Rep.

[44] Rhee MK, Lee YH, Park SH, Sohn CH, Chung YC, Hong SK (1995). A standardization study of Beck Depression Inventory I-Korean Version (K-BDI). Kor J Psychopathol.

[45] Nassir Ghaemi S, Miller CJ, Berv DA, Klugman J, Rosenquist KJ, Pies RW (2005). Sensitivity and specificity of a new bipolar spectrum diagnostic scale. J Affect Disord.

[46] Donovan DM, Kivlahan DR, Doyle SR, Longabaugh R, Greenfield SF (2006). Concurrent validity of the Alcohol Use Disorders Identification Test (AUDIT) and AUDIT zones in defining levels of severity among out-patients with alcohol dependence in the COMBINE study. Addiction.

[47] Saunders JB, Aasland OG, Babor TF, de la Fuente JR, Grant M (1993). Development of the Alcohol Use Disorders Identification Test (AUDIT): WHO Collaborative Project on Early Detection of Persons with Harmful Alcohol Consumption--II. Addiction.

[48] Lee BO, Lee CH, Lee PG, Choi MJ, Kee N (2000). Development of Korean Version of Alcohol Use Disorders Identification Test (AUDIT-K): Its Reliability and Validity. Korean Acad Addiction Psychiatry.

[49] D'Zurilla TJ, Sheedy CF (1991). Relation between social problem-solving ability and subsequent level of psychological stress in college students. J Pers Soc Psychol.

[50] Towbes LC, Towbes LC, Cohen LH (1996). Chronic stress in the lives of college students: Scale development and prospective prediction of distress. J Youth Adol.

[51] Sajatovic M (2005). Bipolar disorder: disease burden. Am J Manag Care.

[52] Krishnan KR (2005). Psychiatric and medical comorbidities of bipolar disorder. Psychosom Med.

[53] Dilsaver SC, Akiskal HS (2005). High rate of unrecognized bipolar mixed states among destitute Hispanic adolescents referred for "major depressive disorder". J Affect Disord.

[54] Murphy GE (1992). Suicide in alcoholism.

[55] Lipschitz A (1995). Suicide prevention in young adults (age 18-30). Suicide Life Threat Behav.

[56] Taylor R, Page A, Morrell S, Harrison J, Carter G (2005). Mental health and socio-economic variations in Australian suicide. Soc Sci Med.

[57] Weissman MM, Bland RC, Canino GJ, Greenwald S, Hwu HG, Joyce PR (1999). Prevalence of suicide ideation and suicide attempts in nine countries. Psychol Med.

[58] Rudd MD (1990). An integrative model of suicidal ideation. Suicide Life Threat Behav.

[59] Dubow EF, Tisak J, Causey D, Hryshko A, Reid G (1991). A two-year longitudinal study of stressful life events, social support, and social problem-solving skills: contributions to children's behavioral and academic adjustment. Child Dev.

[60] Galfalvy HC, Oquendo MA, Mann JJ (2008). Evaluation of clinical prognostic models for suicide attempts after a major depressive episode. Acta Psychiatr Scand.

[61] Beck AT, Brown GK, Steer RA, Dahlsgaard KK, Grisham JR (1999). Suicide ideation at its worst point: a predictor of eventual suicide in psychiatric outpatients. Suicide Life Threat Behav.

[62] Witte TK, Fitzpatrick KK, Joiner TE, Schmidt NB (2005). Variability in suicidal ideation: a better predictor of suicide attempts than intensity or duration of ideation?. J Affect Disord.

